# Distribution of hepatitis C virus in eastern China from 2011 to 2020: a Bayesian spatiotemporal analysis

**DOI:** 10.3389/fpubh.2024.1282575

**Published:** 2024-02-21

**Authors:** Dandan Yang, Chuanfeng Zhang, Yuheng Chen, Jing Lu, Yunting Chen, Zhi Zhang, Feifei Chai, Zhendong Zhang, Furu Wang, Baoli Zhu

**Affiliations:** ^1^Jiangsu Provincial Center for Disease Control and Prevention, Nanjing, China; ^2^Department of Genetic Toxicology, Key Laboratory of Modern Toxicology of the Ministry of Education, Center for Global Health, School of Public Health, Nanjing Medical University, Nanjing, China; ^3^Department of Sexually Transmitted Diseases and AIDS, Xuzhou Center for Disease Control and Prevention, Xuzhou, China

**Keywords:** hepatitis C, HCV, Bayesian model, reported incidence, spatiotemporal distribution

## Abstract

**Objective:**

This study aimed to evaluate the spatiotemporal distribution of patients with hepatitis C virus (HCV) and the factors influencing this distribution in Jiangsu Province, China, from 2011 to 2020.

**Methods:**

The incidence of reported HCV in Jiangsu Province from 2011 to 2020 was obtained from the Chinese Information System for Disease Control and Prevention (CISDCP). R and GeoDa software were used to visualize the spatiotemporal distribution and the spatial autocorrelation of HCV. A Bayesian spatiotemporal model was constructed to explore the spatiotemporal distribution of HCV in Jiangsu Province and to further analyze the factors related to HCV.

**Results:**

A total of 31,778 HCV patients were registered in Jiangsu Province. The registered incidence rate of HCV increased from 2.60/100,000 people in 2011 to 4.96/100,000 people in 2020, an increase of 190.77%. Moran's *I* ranged from 0.099 to 0.354 (*P* < 0.05) from 2011 to 2019, indicating a positive spatial correlation overall. The relative risk (*RR*) of the urbanization rate, the most important factor affecting the spread of HCV in Jiangsu Province, was 1.254 (95% confidence interval: 1.141–1.376), while other factors had no significance.

**Conclusion:**

The reported HCV incidence rate integrally increased in the whole Jiangsu Province, whereas the spatial aggregation of HCV incidence was gradually weakening. Our study highlighted the importance of health education for the floating population and reasonable allocation of medical resources in the future health work.

## 1 Introduction

Hepatitis C is an infectious disease caused by hepatitis C virus (HCV), which is transmitted mainly through blood, sexual contact, and mother-to-fetus transmission (1). HCV is tightly associated with hepatocellular carcinoma (HCC) and it is a long-term process involves a sequence of steps, including chronic hepatic inflammation, liver fibrosis, steatosis, cirrhosis, irreversible genetic or epigenetic alterations, and progression of the malignant carcinogenic cells without effective treatment (2). The World Health Organization (WHO) estimates that approximately 58 million patients with chronic HCV infection (3) and approximately 399,000 people die each year from HCV-related complications (4). In 2016, the WHO Global Health Sector Strategy (GHSS) on viral hepatitis explicitly proposed a target to reduce the incidence of new hepatitis C cases by 90% by 2030 (5). In China, HCV causes a great disease burden, with approximately 7.6 million people living with chronic infections (6), and the incidence of HCV has demonstrated an upward trend (7). Jiangsu Province is one of the most developed provinces in eastern China and has over 80 million permanent residents. The enormous population size and population mobility poses a great challenge for HCV prevention and control, although Jiangsu Province is an area with low endemicity for HCV infection.

Previous studies showed that HCV prevalence is influenced by spatial and temporal factors (8, 9). An HCV epidemiological survey showed that there were obvious regional differences in HCV incidence in China. Some researchers established 11 provinces as hotspots of clustered HCV-infected patients by performing a spatial analysis of HCV in mainland China from 2005 to 2011 (10). Zhu et al. (11) reported that the epidemic of HCV in China became much more severe from 2003 to 2015, and the hotspots gradually shifted from northeastern to western China. The hotspots identified in these studies were mostly located in economically underdeveloped areas or border areas of China. The incidence of HCV in rural areas is significantly higher than that in urban areas, and the incidence in the central and western regions is higher than that in the eastern regions. The larger cluster of HCV-infected patients in these areas is attributed to poorer economic status or drug use. However, for Jiangsu Province, an economically developed province in the eastern region of China, no study has clarified the spatiotemporal epidemiological characteristics of HCV. The Bayesian spatiotemporal model provides a robust method to integrate embedded temporal information, spatial information, and parameter uncertainty (prior distribution) (12), thereby describing the spatiotemporal characteristics and identifying spatiotemporally associated factors. In this study, we used the Bayesian spatiotemporal model to investigate the spatiotemporal distribution of HCV in Jiangsu Province from 2011 to 2020 and to further explore the factors related to HCV incidence.

## 2 Materials and methods

### 2.1 Data sources

The incidence data on registered HCV patients from 2011 to 2020 were obtained from the Chinese Information System for Disease Control and Prevention (CISDCP) of Jiangsu Province, and analysis was conducted according to the current address of the patients. The number of permanent residents was calculated as the number of HCV patients in an area divided by the incidence in different years. Other demographic and socioeconomic variables, including the urbanization rate, disposable income per capita, number of doctors, number of beds per 1,000 people, and national basic medical insurance participation rate, were collected from the Statistical Yearbook (13–25) of 13 cities in Jiangsu Province from 2011 to 2020. The vector maps of counties in Jiangsu Province were obtained from the database of the China National Catalog Service for Geographic Information (https://www.webmap.cn/main.do?method=index).

### 2.2 Spatial autocorrelation analysis

Spatial autocorrelation analysis was performed at the district and county levels. Global spatial autocorrelation was used to analyze the overall spatial aggregation of the HCV epidemic in ninety-six districts and counties of Jiangsu Province. Moran's *I* was set between [−1, 1] and was calculated as follows Equation 1 (26):


(1) $$\begin{array}{l} I=\frac{n\sum_{i=1}^{n}\sum_{j=1}^{n}w_{ij}\left(x_{i}-\bar{x}\right)\left(x_{j}-\bar{x}\right)}{\left(\sum_{i=1}^{n}\sum_{j=1}^{n}w_{ij}\right)\sum_{i=1}^{n}{\left(x_{i}-\bar{x}\right)}^{2}}\;,\;i=j \end{array}$$

where *n* are the number of districts and counties in Jiangsu Province, *x*_*i*_ and *x*_*j*_ are the hepatitis C patients in districts *i* and *j*, respectively, $\bar{x}$ is the average number of registered hepatitis C patients in all regions in this study, and *w*_*ij*_ represents the adjacent weight matrix corresponding to the district pair *i* and *j*. If *p* < 0.05, the spatial correlation was considered statistically significant. Moran's *I* > 0 indicated a positive spatial correlation, whereas Moran's *I* < 0 indicated a negative correlation. Local indicators of spatial association (LISA) analysis was conducted to estimate the impact of individual areas on the overall situation and types of clusters. All the above analyses were performed by GeoDa (version 1.18.0.0; Center for Spatial Data Science).

### 2.3 Bayesian spatiotemporal model analysis

We studied the impact of the urbanization rate, disposable income per capita, number of doctors and beds, and on the registered HCV incidence for each city in Jiangsu Province from 2011 to 2020. The Bayesian spatiotemporal model is a hierarchical model based on Markov chain Monte Carlo (MCMC) simulations (12). The number of HCV patients was generally considered to follow a Poisson distribution as follows Equation 2 (27):


(2) $$\begin{array}{l} y_{it}\sim Poisson\left(E_{it}\theta_{it}\right) \end{array}$$

where *y*_*it*_ represents the registered HCV patient number in the *t*^th^ year (*t* = 1, 2, …, 10) and region *i* (*i* = 1, 2, …, 13), *E*_*it*_ is the expected number of HCV patients in year *t* and region *i*, and θ_*it*_ is the ratio of the actual number of HCV patients to the expected number in year *t* and region *i*, which represents the relative risk (*RR)* of disease incidence. A Bayesian model was constructed based on the logarithmic form of θ_*it*_, computed as follows Equation 3:


(3) $$\begin{array}{l} \log\left(\theta_{it}\right)=\beta_{0}+\overset{5}{\underset{k=1}{\sum }}x_{ik}\beta_{k}+u_{i}+v_{t} \end{array}$$

β_0_ is the intercept, β_*k*_ denotes the regression coefficients of the collected factors, and *x*_*i*1_, …, *x*_*i*5_ represent the urbanization rate, disposable income per capita, number of doctors and beds per 1,000 people, and national basic medical insurance participation rate, respectively.

In Equation 4, *u*_*i*_ is the spatial structure effect, reflecting spatial correlation, which is assumed to subject to a conditional autoregressive process, with a Gaussian distribution, and the mean being the weighted average of neighboring regions *u*_*j*_, *i* ≠ *j*, computed as follows, where δ_*i*_ is the first-order neighborhood of region *i*, *n*_δ_*i*__ is the number of neighboring regions in region *i*, and $\sigma_{e}^{2}$ is the variance of the spatial effect.


(4) $$\begin{array}{l} \;u_{i}|u_{j},\;i\neq j\sim N\left(\underset{j\in \delta_{i}}{\sum }\frac{u_{j}}{n_{\delta_{i}}},\frac{\sigma_{e}^{2}}{n_{\delta_{i}}}\right) \end{array}$$

*v*_*t*_ is the temporal structure effect, whose prior distribution is a first-order general autoregressive (AR) model (1), reflecting that the temporal effect *v*_*t*_ at moment *t* is only related to the temporal effect *v*_*t*−1_ at the previous moment. The above model was analyzed by the CARBayesST package in R (version 3.6.2; R Foundation for Statistical Computing).

## 3 Results

### 3.1 Spatiotemporal distribution of HCV incidence in Jiangsu Province

**Figure 1:**
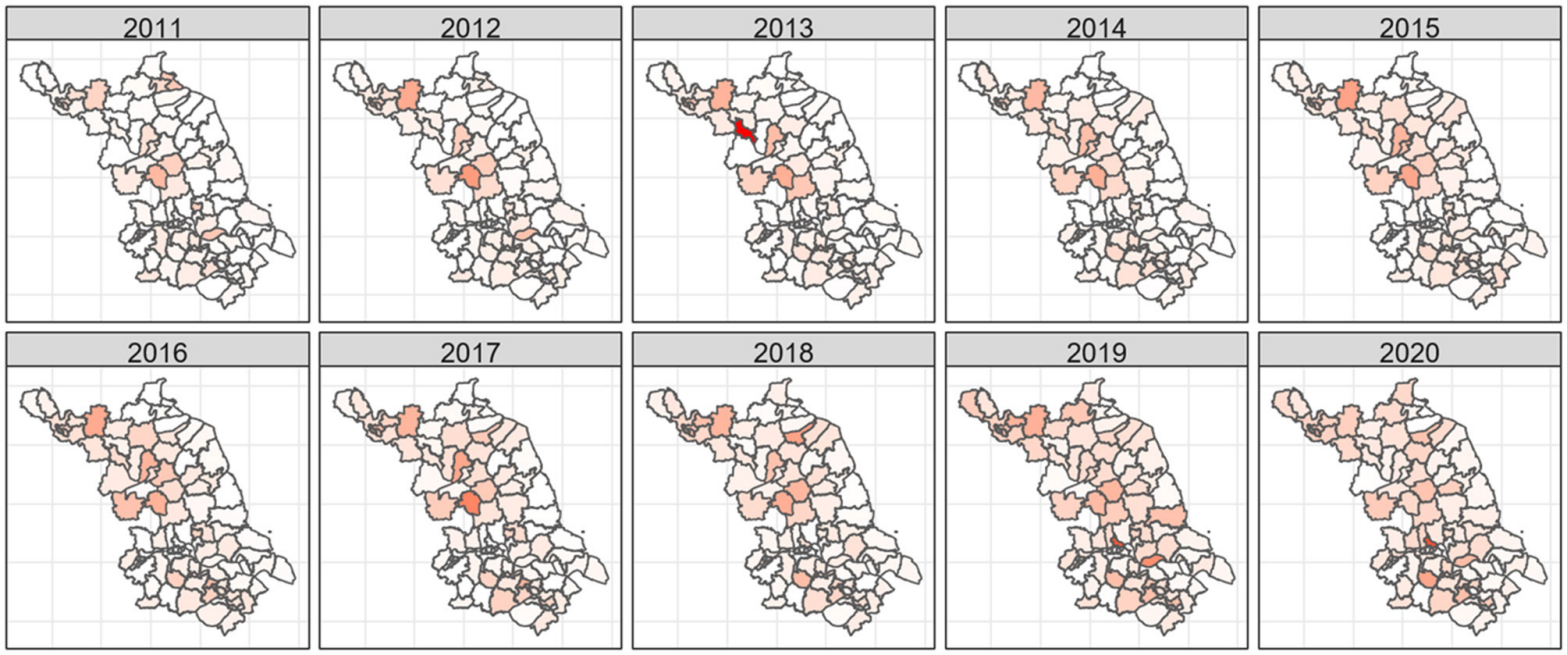
Reported HCV incidence by district in Jiangsu Province from 2011–2020.

A total of 31,778 HCV patients were registered in Jiangsu Province from 2011 to 2020. The registered incidence of HCV rose from 2.60 patients per 100,000 people in 2011 to 4.96 patients per 100,000 in 2020, an increase of 190.77%, with the highest incidence in 2019 (5.27 patients per 100,000 people). As shown in Table 1, Huai'an city (6.86 patients per 100,000 people) and Xuzhou city (6.62 patients per 100,000 people) located in the northern area of Jiangsu Province and Wuxi city (5.44 patients per 100,000 people) located in the southern area were the regions with the highest average incidence of HCV. In contrast, Nantong and Nanjing city situated in the south of Jiangsu Province had the lowest incidence. In general, the northern region of Jiangsu Province had a higher HCV incidence, which increased over time. Figure 1 shows the change in the reported incidence of HCV in Jiangsu Province from 2011–2020; the darker the color is the higher the HCV incidence.

**Table 1:** Number of HCV cases and incidence rates by city in Jiangsu Province from 2011 to 2020.

**Year**	**Cases per city, n/N (per 100,000 people)**												
	**Nanjing**	**Wuxi**	**Xuzhou**	**Changzhou**	**Suzhou**	**Nantong**	**Lianyungang**	**Huai-an**	**Yancheng**	**Yangzhou**	**Zhenjiang**	**Taizhou**	**Suqian**
2011	201/ 8,109,100 (2.48)	185/ 6,432,200 (2.88)	331/ 8,572,600 (3.86)	158/ 4,649,700 (3.40)	177/ 10,518,700 (1.68)	67/ 7,289,100 (0.92)	142/ 4,386,100 (3.24)	208/ 4,803,400 (4.33)	99/ 7,237,400 (1.37)	152/ 4,463,000 (3.41)	92/ 3,134,300 (2.94)	177/ 4,626,000 (3.83)	67/ 4,766,400 (1.41)
2012	167/ 8,161,000 (2.05)	203/ 6,465,500 (3.14)	509/ 8,564,100 (5.94)	190/ 4,686,800 (4.05)	255/ 10,549,100 (2.42)	94/ 7,297,300 (1.29)	72/ 4,406,900 (1.63)	276/ 4,803,000 (5.75)	110/ 7,216,300 (1.52)	168/ 4,467,200 (3.76)	79/ 3,154,800 (2.50)	201/ 4,629,800 (4.34)	97/ 4,798,000 (2.02)
2013	182/ 8,187,800 (2.22)	302/ 6,484,100 (4.66)	536/ 8,591,000 (6.24)	227/ 4,692,100 (4.84)	266/ 10,578,700 (2.51)	68/ 7,297,700 (0.93)	57/ 4,428,300 (1.29)	291/ 4,826,900 (6.03)	125/ 7,219,800 (1.73)	161/ 4,470,000 (3.60)	74/ 3,165,400 (2.34)	133/ 4,634,000 (2.87)	372/ 4,819,100 (7.72)
2014	150/ 8,216,100 (1.83)	322/ 6,500,100 (4.95)	497/ 8,628,300 (5.76)	239/ 4,696,400 (5.09)	250/ 10,604,000 (2.36)	108/ 7,298,000 (1.48)	53/ 4,451,700 (1.19)	360/ 4,852,100 (7.42)	158/ 7,222,800 (2.19)	157/ 4,477,900 (3.51)	87/ 3,171,400 (2.74)	155/ 4,638,600 (3.34)	198/ 4,843,200 (4.09)
2015	136/ 8,235,900 (1.65)	339/ 6,511,000 (5.21)	602/ 8,669,000 (6.94)	238/ 4,701,400 (5.06)	341/ 10,616,000 (3.21)	144/ 7,300,000 (1.97)	71/ 4,473,700 (1.59)	412/ 4,872,000 (8.46)	230/ 7,228,500 (3.18)	156/ 4,483,600 (3.48)	85/ 3,176,500 (2.68)	179/ 4641600 (3.86)	226/ 4,853,800 (4.66)
2016	132/ 8,270,000 (1.60)	458/ 6,529,000 (7.01)	629/ 8,710,000 (7.22)	203/ 4,708,300 (4.31)	337/ 10,647,400 (3.17)	183/ 7,302,000 (2.51)	67/ 4,496,400 (1.49)	373/ 4,890,000 (7.63)	183/ 7,235,000 (2.53)	137/ 4,491,400 (3.05)	76/ 3,181,300 (2.39)	177/ 4,645,800 (3.81)	274/ 4,879,400 (5.62)
2017	115/ 8,335,000 (1.38)	468/ 6,553,000 (7.14)	607/ 8,763,500 (6.93)	206/ 4,717,300 (4.37)	332/ 10,683,600 (3.11)	171/ 7,305,000 (2.34)	140/ 4,518,400 (3.10)	406/ 4,914,000 (8.26)	195/ 7,242,200 (2.69)	153/ 4,508,200 (3.39)	60/ 3,186,300 (1.88)	206/ 4,651,900 (4.43)	231/ 4,914,600 (4.70)
2018	176/ 8,436,200 (2.09)	390/ 6,574,500 (5.93)	656/ 8,802,000 (7.45)	220/ 4,728,600 (4.65)	326/ 10,721,700 (3.04)	224/ 7,310,000 (3.06)	195/ 4,520,000 (4.31)	325/ 4,925,000 (6.60)	245/ 7,200,000 (3.40)	241/ 4,531,000 (5.32)	84/ 3,196,400 (2.63)	162/ 4635700 (3.49)	178/ 4,925,900 (3.61)
2019	265/ 9,281,600 (2.86)	444/ 7,453,600 (5.96)	801/ 9,062,800 (8.84)	240/ 5,272,500 (4.55)	425/ 12,707,500 (3.34)	261/ 7,724,400 (3.38)	242/ 4,590,900 (5.27)	342/ 4,640,900 (7.37)	375/ 6,708,400 (5.59)	394/ 4,549,000 (8.66)	80/ 3,203,500 (2.50)	384/ 4,523,000 (8.49)	212/ 4,972,800 (4.26)
2020	326/ 9,319,700 (3.50)	532/ 7,464,000 (7.13)	625/ 9,083,900 (6.88)	286/ 5,279,600 (5.42)	358/ 12,749,600 (2.81)	254/ 7,728,000 (3.29)	239/ 4,601,000 (5.19)	305/ 45,59,200 (6.69)	279/ 6,710,600 (4.16)	436/ 4,561,000 (9.56)	104/ 3,211,000 (3.24)	261/ 4,516,800 (5.78)	203/ 4,988,200 (4.07)
Total	1850/ 84,552,400 (2.19)	3643/ 66,967,000 (5.44)	5793/ 8,744,7200 (6.62)	2207/ 48,132,700 (4.59)	3067/ 110,376,300 (2.78)	1574/ 73,851,500 (2.13)	1278/ 44,873,400 (2.85)	3298/ 48,086,500 (6.86)	1999/ 71,221,000 (2.81)	2155/ 45,002,300 (4.79)	821/ 31,780,900 (2.58)	2035/ 46,143,200 (4.41)	2058/ 48,761,400 (4.22)

### 3.2 Spatial autocorrelation analysis of HCV incidence

**Figure 2:**
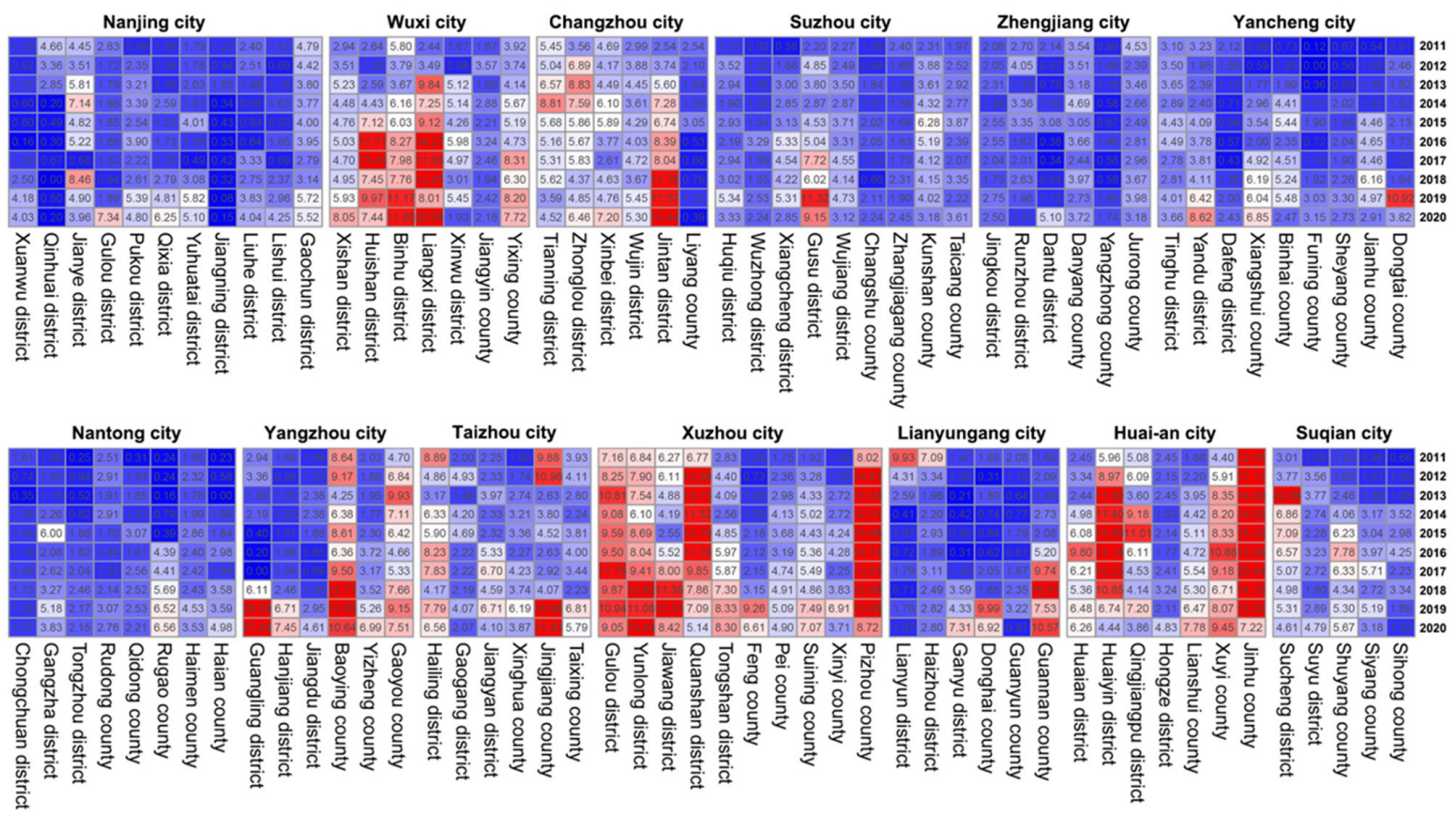
Heatmap of reported HCV incidence by district in Jiangsu Province from 2011 to 2020.

**Figure 3:**
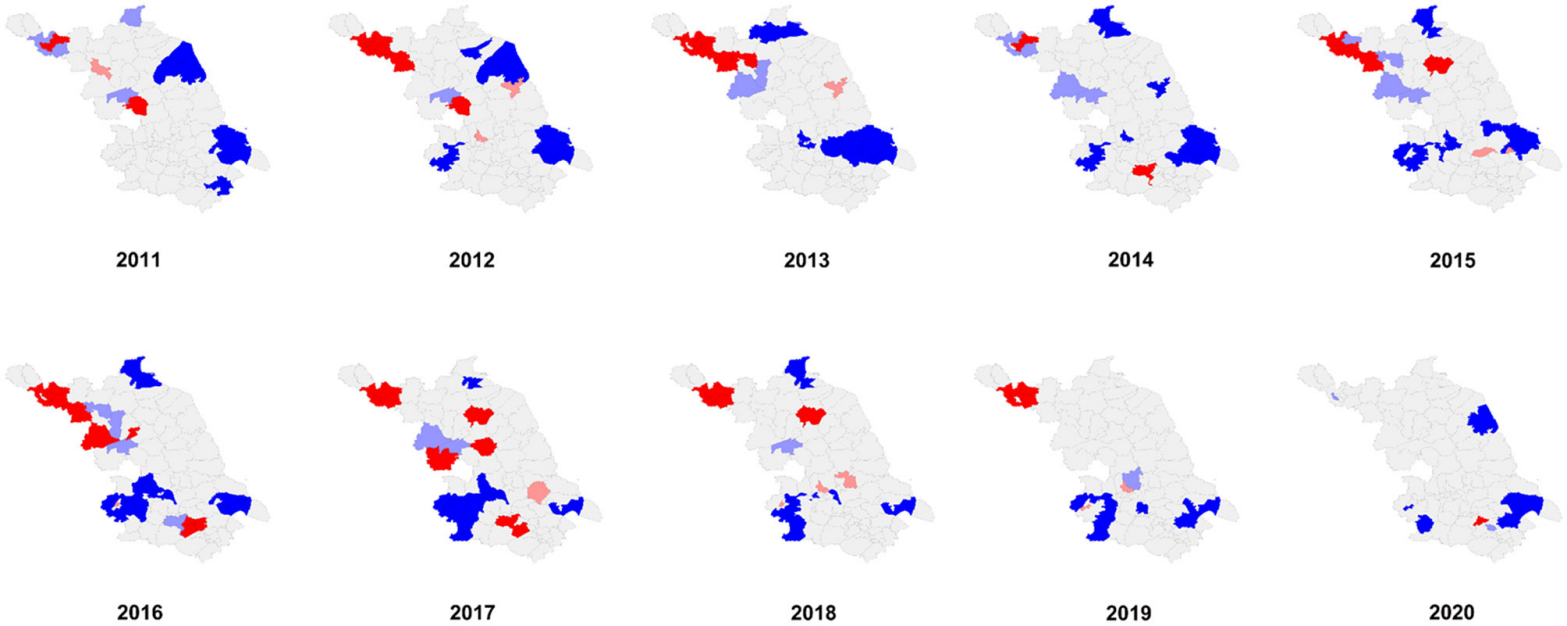
LISA cluster map of HCV incidence in Jiangsu Province from 2011 to 2020.

We performed spatial autocorrelation analysis at the district and county levels. The specific registered HCV incidence at the district and county levels is shown in a heatmap (Figure 2). The results of the global spatial autocorrelation analysis of HCV incidence over the last decade are described in Table 2. Moran's *I* ranged from 0.099 to 0.354 (*P* < 0.05 for each Moran's *I*) from 2011 to 2019, suggesting a positive spatial correlation of HCV incidence at the district and county levels in Jiangsu Province. Moran's *I* in 2020 was 0.073 (*P* > 0.05), indicating that there was no spatial clustering and that the incidence of HCV was randomly distributed throughout the province. Before 2013, the hotspots of HCV incidence were mainly located in some districts or counties belonging to Xuzhou city and Huai'an city in the northern region of Jiangsu Province (Figure 3). After 2014, the hotspots gradually spread from the northern to the southern region. The coldspots of HCV incidence were consistently located in Nanjing city and Nantong city during the study period (Figure 3).

**Table 2:** Results of global spatial autocorrelation analysis of HCV incidence in Jiangsu Province, China, from 2011 to 2020.

**Year**	**Moran's *I***	***E(I)***	***Z* value**	***P* value**
2011	0.191	−0.0105	3.1471	0.003
2012	0.265	−0.0105	4.4015	<0.001
2013	0.143	−0.0105	2.7165	0.012
2014	0.298	−0.0105	4.7894	<0.001
2015	0.279	−0.0105	4.5237	<0.001
2016	0.354	−0.0105	5.6826	<0.001
2017	0.351	−0.0105	5.7112	<0.001
2018	0.286	−0.0105	4.6207	<0.001
2019	0.099	−0.0105	1.7469	0.049
2020	0.073	−0.0105	1.3521	0.092

### 3.3 Bayesian analysis of registered HCV patients in Jiangsu Province

Bayesian spatiotemporal model analysis revealed that the urbanization rate was the most important factor affecting the epidemic situation of HCV in Jiangsu Province [*RR* = 1.254, 95% confidence interval (*CI*): 1.141–1.376], while other factors had no significant effect on the risk of HCV incidence (Table 3). The *RR* of HCV infection increased by 25.4% for every 1% increase in the urbanization rate.

**Table 3:** Bayesian spatiotemporal model regression coefficient values.

**Variable**	**Median (95% *CI*)**	**Posteriori estimated relative risk values (95% *CI*)**
Urbanization rate	0.0252 (0.0146 to 0.0352)	1.254 (1.141 to 1.376)
Disposable income per capita	−0.0154 (−0.0371 to 0.0074)	0.904 (0.782 to 1.047)
Number of doctors	0.0383 (−0.0421 to 0.1271)	1.032 (0.966 to 1.105)
Number of beds	−0.0914 (−0.2924 to 0.1037)	0.954 (0.852 to 1.064)
Basic medical insurance participation rate	0.0009 (−0.0008 to 0.0027)	1.018 (0.985 to 1.055)
τ^2^	0.0167 (0.0100 to 0.0264)	
ρ_*S*_	0.3401 (0.0571 to 0.7055)	
ρ_*T*_	0.9804 (0.9289 to 0.9990)	

τ^2^ is the process variance; ρ_*S*_ is the level of spatial autocorrelation; ρ_*T*_ is the level of temporal autocorrelation.

## 4 Discussion

This study analyzed the spatial and temporal characteristics of the incidence of HCV in Jiangsu Province. We found that the incidence of HCV generally increased but varied widely by region from 2011 to 2020 in Jiangsu Province. Based on the Bayesian spatiotemporal model, the urbanization rate was the most important factor affecting HCV incidence in Jiangsu Province.

In China, blood transfusion, especially transfusion of contaminated blood or blood products, was the dominant mode of HCV transmission in the late 1980s and early 1990s (1). In 1998, the Chinese government outlawed paid blood donations (28) for clinical use, drastically reducing transmission from blood or blood products. However, due to the lack of medical resources or health awareness, many people did not receive the test for HCV infection, which became a significant barrier to achieving HCV elimination (29). Fortunately, the expansion of insurance coverage and patient reimbursement schemes in China resulted in the increased exposure of large segments of the population to the health care system. Active or passive medical screening became more frequent with age in these populations that were once exposed to contaminated blood or blood products, leading to an increase in HCV detection rates. In contrast to the past, injecting drug users (IDUs) and sexual transmission are no the main routes of HCV transmission (30). IDUs and unsafe sex activities also contributed to the spread of HCV from high-risk populations to the general population. In general, IDUs and sexual transmission might be responsible for the upward trend of HCV in Jiangsu Province.

Spatial autocorrelation analysis indicated that HCV clusters had significant spatial aggregation before 2020 in Jiangsu Province. Before 2013, HCV hotspots were mainly located in the cities of Xuzhou and Huai'an in the northern region of Jiangsu Province. After 2014, the southern region of Jiangsu Province also appeared as a hotspot, such as Wujin District in Changzhou city and Binhu and Liangxi districts in Wuxi city. Hotspots gradually spread from the northern to the southern regions of Jiangsu Province. Remarkably, Xuzhou city was a highly clustered area of HCV prevalence from 2011 to 2019 in Jiangsu Province. Geographical location is one of the major reasons for the high HCV prevalence in Xuzhou city. Xuzhou city is located at the border of four provinces, including Henan Province, a region with a severe HCV epidemic due to illegal blood donations. The complicated human mobility situation and relatively unfavorable economic conditions aggravate the spread of HCV in Xuzhou city. The southern areas in Jiangsu Province are highly developed and can offer great employment opportunities, which lead to the migration of many workers to these regions. In addition, according to the sentinel surveillance from the Jiangsu Provincial Centers for Disease Control and Prevention, the population size of IDUs and people with HIV in southern Jiangsu is significantly larger than that in northern Jiangsu. As a result, population mobility and a higher proportion of IDUs and unprotected sexual encounters might be responsible for the spread of hotspots from the northern to the southern regions of Jiangsu Province.

This study found that the urbanization rate was the most important factor affecting the epidemic situation of HCV in Jiangsu. The *RR* of HCV infection increased by 25.4% for every 1% increase in the urbanization rate. In contrast, other factors had no significant effect on the risk of hepatitis C in this model. Continuing rural-to-urban migration with the development of urbanization swelled the population density of the city, thus inevitably exacerbating the intensified transmission risk of HCV. Therefore, during the process of urbanization, reasonable allocation of medical resources should be highly emphasized to ensure that the health service needs of various types of floating populations in cities are met.

Due to insoluble constraints, this study still has some limitations. The most important limitation is that the demographic and socioeconomic information was obtained from the Statistical Yearbook at the prefectural level, and some factors affecting the incidence of hepatitis C may have been concealed. Further research is needed in the future. In addition, data on HCV patients and incidence relied on the CISDCP. Due to the discrepant quality of reports in different regions, there may have been discrepancies between the reported number of HCV patients and the real situation.

## 5 Conclusion

In conclusion, the reported incidence of HCV in Jiangsu Province showed a significant upward trend from 2011 to 2020, whereas the spatial aggregation of HCV incidence was gradually weaking. Rising urbanization rates was a crucial factor affecting the incidence of HCV, therefore, our study highlighted the importance of health education for the floating population and reasonable allocation of medical resources in the future health work.

## Data availability statement

The raw data supporting the conclusions of this article will be made available by the authors, without undue reservation.

## Author contributions

DY: Writing—original draft. CZ: Writing—original draft. YuhC: Writing—review & editing. JL: Writing—original draft. YunC: Writing—review & editing. ZhiZ: Writing—review & editing. FC: Writing—review & editing. ZheZ: Project administration, Supervision, Writing—review & editing. FW: Methodology, Writing—review & editing. BZ: Project administration, Supervision, Writing—review & editing.
