# Supplementary material for: Distribution of hepatitis C virus in eastern China from 2011 to 2020: a Bayesian spatiotemporal analysis
Source: Front Public Health. 2024 Feb 21;12:1282575. doi: 10.3389/fpubh.2024.1282575 (PMC10914966; doi:10.3389/fpubh.2024.1282575)
Supplement: Supplementary file 1 [file Data_Sheet_1.PDF]

```
dat <- read.csv(file="modeldat22.csv")
head(dat,n=3)

library(rgdal)
library(ggplot2)
library(maptools)
library(mapproj)
library(ggsn)
JS <- sf::st_read("jscitymap.shp")

#### Fit a simple Poisson log-linear model
model1 <- glm(formula=Y~offset(log(E))+urbanrate+income+bed+physician+insurance,
                    family="poisson", data=dat)
round(cbind(model1$coefficients, confint(model1)),4)

#### compute the residuals from this model
library(dplyr)
library(leaflet)
dat$residuals <- residuals(model1)
residuals2014 <- filter(dat, year==2014)
residuals2014.JS <- merge(x=JS, y=residuals2014, by.x="市代码", by.y="市代码", all.x=FALSE)

#### Construct the spatial objects
library(spdep)
library(sf)
W.nb <- poly2nb(residuals2014.JS, row.names = residuals2014.JS$"市代码")
W <- nb2mat(W.nb, style = "B")
W.list <- nb2listw(W.nb, style = "B")
moran.mc(x = residuals2014.JS$residuals, listw = W.list, nsim = 10000)

#### Order the data according to the neighbourhood matrix and year
lookup <- data.frame(Code=residuals2014.JS$"市代码", spatialorder=1:nrow(residuals2014.JS))
dat.temp <- merge(x=dat, y=lookup, by.x="市代码", by.y="Code")
dat.ordered <- arrange(dat.temp, year, spatialorder)

#### Fit the model
library(CARBayesST)
library(CARBayes)


chain1    <-    ST.CARar(formula=Y~offset(log(E))+urbanrate+income+bed+physician+insurance,
family="poisson", data=dat.ordered, W=W,
                              burnin=200000, n.sample=2200000, thin=1000, verbose=FALSE,AR=1)
chain2    <-    ST.CARar(formula=Y~offset(log(E))+urbanrate+income+bed+physician+insurance,
```

```
family="poisson", data=dat.ordered, W=W,
                    burnin=200000, n.sample=2200000, thin=1000, verbose=FALSE,AR=1)
chain3 <- ST.CARar(formula=Y~offset(log(E))+urbanrate+income+bed+physician+insurance,
family="poisson", data=dat.ordered, W=W,
                    burnin=200000, n.sample=2200000, thin=1000, verbose=FALSE,AR=1)

#### Check convergence - traceplot
library(coda)
beta.samples <- mcmc.list(chain1$samples$beta, chain2$samples$beta, chain3$samples$beta)


#### Check convergence - Gelman-Rubin plot
gelman.diag(beta.samples)

#### Model summary
print(chain1)



#### Effects of covariates on disease risk
sd(dat.ordered$urbanrate)
sd(dat.ordered$income)
sd(dat.ordered$bed)
sd(dat.ordered$physician)
sd(dat.ordered$insurance)

beta.samples.combined<- rbind(chain1$samples$beta, chain2$samples$beta, chain3$samples$beta)
round(quantile(exp(sd(dat.ordered$urbanrate) * beta.samples.combined[ ,2]), c(0.5, 0.025,
0.975)),3)
round(quantile(exp(sd(dat.ordered$income) * beta.samples.combined[ ,3]), c(0.5, 0.025, 0.975)),3)
round(quantile(exp(sd(dat.ordered$bed) * beta.samples.combined[ ,4]), c(0.5, 0.025, 0.975)),3)
round(quantile(exp(sd(dat.ordered$physician) * beta.samples.combined[ ,5]), c(0.5, 0.025,
0.975)),3)
round(quantile(exp(sd(dat.ordered$insurance) * beta.samples.combined[ ,6]), c(0.5, 0.025,
0.975)),3)

fitted.samples.combined<-
rbind(chain1$samples$fitted,chain2$samples$fitted,chain3$samples$fitted)
n.samples<-nrow(fitted.samples.combined)
n.all<-ncol(fitted.samples.combined)
risk.samples.combined<-fitted.samples.combined/matrix(
  rep(dat.ordered$E,n.samples),nrow = n.samples,ncol = n.all,byrow = TRUE)
```
